# A favorable cardiometabolic profile is associated with the G allele of the genetic variant rs5068 in African Americans: The Multi-Ethnic Study of Atherosclerosis (MESA)

**DOI:** 10.1371/journal.pone.0189858

**Published:** 2017-12-18

**Authors:** Valentina Cannone, Christopher G. Scott, Paul A. Decker, Nicholas B. Larson, Walter Palmas, Kent D. Taylor, Thomas J. Wang, Deepak K. Gupta, Suzette J. Bielinski, John C. Burnett

**Affiliations:** 1 Cardiorenal Research Laboratory, Mayo Clinic, Rochester, Minnesota, United States of America; 2 Division of Clinical Medicine, Department of Medicine and Surgery, University of Parma, Parma, Italy; 3 Department of Health Sciences Research, Mayo Clinic, Rochester, Minnesota, United States of America; 4 Department of Medicine Columbia University College of Physicians and Surgeons, Columbia University, New York, New York, United States of America; 5 Los Angeles Biomedical Research Institute (LA BioMed), Institute for Translational Genomics and Population Sciences, Harbor-UCLA Medical Center, Torrance, California, United States of America; 6 Vanderbilt Translational and Clinical Cardiovascular Research Center and Division of Cardiovascular Medicine, Vanderbilt University Medical Center, Nashville, Tennessee, United States of America; Virgen Macarena University Hospital, School of Medicine, University of Seville, SPAIN

## Abstract

In whites, the minor G allele of the atrial natriuretic peptide (ANP) genetic variant rs5068 is associated with higher circulating levels of ANP and B-type natriuretic peptide (BNP), lower risk of hypertension, higher high-density lipoprotein (HDL) cholesterol plasma levels, and lower prevalence of obesity and metabolic syndrome. The observed phenotype is consistent with the blood pressure lowering and metabolic properties of ANP and BNP. The cardiovascular and metabolic phenotype associated with rs5068 genotypes in African Americans is undefined. We genotyped 1631 African Americans in the Multi-Ethnic Study of Atherosclerosis (MESA) for rs5068 and investigated their phenotype. Genotype frequencies of rs5068 were 93.2% AA (n = 1520), 6.7% AG (n = 110) and 0.1% GG (n = 1). All subsequent analyses are AG + GG versus AA genotype. Using a Bonferroni corrected level of significance of 0.005, the prevalence of metabolic syndrome (23% vs 38%, age-sex-adjusted p = 0.002) and triglycerides plasma values (76 vs 90 mg/dl, age-sex-BMI adjusted p = 0.004) were both significantly lower in the AG+GG genotypes. In the AG+GG genotypes, the prevalence of diabetes (8% vs 18%, age-sex-BMI-adjusted p = 0.02) and insulin plasma levels tended to be lower (4.8 vs 5.7 μU/ml, age-sex-BMI adjusted p = 0.04) whereas HDL-cholesterol levels tended to be higher (55 vs 50 mg/dl, age-sex-BMI-adjusted p = 0.04). No association was found with hypertension. The association between the rs5068 G allele and a favorable metabolic phenotype is now shown in African Americans. The rs5068 AG+GG genotypes are associated with lower prevalence of metabolic syndrome and lower triglycerides values.

## Introduction

The natriuretic peptides (NP) atrial natriuretic peptide (ANP) and b-type natriuretic peptide (BNP) are cardiac hormones with well-known blood pressure lowering and natriuretic properties.[1] Increasing recent evidence strongly supports a biological action of ANP and BNP in mediating favorable metabolic actions in adipocytes, muscle cells and animal models via activation of the particulate guanylyl cyclase A receptor (GC-A) and the second messenger cyclic guanosine monophosphate (cGMP). More specifically, studies have shown that in the presence of high-fat diet, overexpression of BNP prevents mice from developing obesity and insulin resistance by promoting muscle mitochondrial biogenesis and fat oxidation.[2] Further, Bordicchia and co-workers reported that NP favor browning of white adipocytes inducing thermogenic energy expenditure.[3] Importantly, in human adipocytes, insulin may up-regulate the NP clearance receptor that would increase the clearance of circulating ANP and BNP and reduce their availability and metabolic actions.[4] The single nucleotide polymorphism rs5068 is a genetic variant of the ANP gene (*NPPA*) which includes the AA, AG and GG genotypes with the G allele being the minor allele. Rs5068 G allele is associated with higher circulating levels of ANP and BNP, and, consequently, the clinical phenotype associated with rs5068 G allele might reveal the cardiovascular and metabolic effect of a long term exposure to slightly higher circulating levels of NP in humans.[5–8] In a study by Newton-Cheh et al, the minor G allele of rs5068 was associated with higher circulating levels of ANP and BNP, lower values of systolic and diastolic blood pressure, and lower odds of hypertension in community-based cohorts from Framingham (MA), Sweden, and Finland.[5] Cannone et al recently reported that in a random sample of the general population from Olmsted County, MN, which included mostly whites, the minor allele of rs5068 was associated with higher plasma ANP levels, lower systolic blood pressure, lower body mass index (BMI) and smaller waist circumference.[6] Carriers of the G allele also had significantly higher plasma values of high-density lipoprotein (HDL)-cholesterol. Moreover, the prevalence of obesity and metabolic syndrome was lower in the carriers of the minor G allele. Importantly, the associations between the minor G allele and a favorable cardiometabolic phenotype were replicated by Cannone et al in a community-based cohort from the Mediterranean city of Ventimiglia di Sicilia, Italy.[7] In a large cohort of Scandinavian subjects, the rs5068 G allele was also associated with lower risk to develop diabetes mellitus in a 14-year follow-up analysis.[9] Therefore, the cardiovascular and metabolic phenotype associated with rs5068 minor allele is consistent with the blood pressure lowering, lipolytic and insulin sensitivity enhancing properties of ANP and BNP via the GC-A.[1, 10, 11]

African Americans are at higher risk for cardiovascular and metabolic disease as compared to other ethnic groups.[12] Thus, a high priority is to understand mechanisms of risk and risk prevention. Recently, racial differences in circulating ANP and BNP levels have been identified in community-based cohorts which show that black individuals have significantly lower circulating NP levels compared with subjects of European ancestry.[13, 14] Relative NP deficiency might be a mechanism contributing to the higher prevalence of hypertension, obesity, diabetes mellitus and metabolic syndrome in African Americans.[15, 16] To date, the phenotype associated with the genotypes of the ANP genetic variant rs5068 has been characterized only in white cohorts while it remains unexplored in African Americans.

The current study analyzed for the first time the cardiovascular and metabolic phenotype associated with rs5068 genotypes in the general community-based cohort of African Americans participating in the Multi-Ethnic Study of Atherosclerosis (MESA), such cohort includes subjects residing in five different geographic locations in the United States. These findings are noteworthy as they provide further insights into the cardiometabolic risk in African Americans and might have implications for a NP-based therapeutics.

## Methods

MESA is a multicenter study of 6,814 subjects (age 45 to 84 years, 3,601 women). The cohort includes individuals who self-identified as non-Hispanic white (38%), African American (28%), Hispanic (22%), or Chinese American (12%). Participants were enrolled from six U.S. field centers: Forsyth County, North Carolina (Wake Forest University School of Medicine); St. Paul, Minnesota (University of Minnesota); Chicago, Illinois (Northwestern University, University of Illinois and Loyola University); New York, New York (Columbia University and St. Francis Hospital); Baltimore, Maryland (Johns Hopkins University) and Los Angeles, California (University of California, Los Angeles). All individuals were free of clinical cardiovascular disease at the time of enrollment. Details regarding study recruitment and design have been previously reported.[17] The study was approved by the Mayo Clinic Institutional Review Board (Approval number: PR09-000633-08) and all participants gave written informed consent. In this study, we analyzed a cohort of 1,631 African Americans enrolled from five of the six field centers (Minnesota did not recruit any African Americans). Subjects were genotyped for rs5068 using the Illumina CardioMetaboChip. For the current study we analyzed the data collected at exam 1 (from 2000 to 2002). Median follow-up was 11 years (interquartile range: 10.5 to 11.5 years). Body mass index was calculated as weight (kg)/ height^2^ (m^2^). Obesity and overweight were defined as BMI ≥ 30 kg/m^2^ and 25 ≤ BMI <30 kg/m^2^, respectively. Blood pressure was measured three times in the seated position using a Dinamap model Pro 100 automated oscillometric sphygmomanometer (Critikon, Tampa, Florida). The average of the last two measurements was used in analysis. Hypertension was defined as systolic blood pressure ≥ 140 mm Hg and/or diastolic blood pressure ≥ 90 mmHg and/or use of antihypertensive medications. Metabolic syndrome was defined according to the National Cholesterol Education Program III Guidelines.[18] Diabetes mellitus was diagnosed based on fasting glucose ≥ 126mg/dL or the use of insulin or oral hypoglycemic medications. In our study cohort (n = 1631), diabetes mellitus status was known in 1626 subjects. Blood lipids, insulin and glucose were measured in blood samples after 12 hours of fasting.[19] Triglycerides, total cholesterol, and HDL-cholesterol concentrations were measured using the cholesterol oxidase method (Roche Diagnostics, Indianapolis, IN) and low density lipoprotein cholesterol plasma values were estimated according to the Friedewald equation.[20] Serum glucose and insulin were measured by the Vitros analyzer (Johnson & Johnson Clinical Diagnostics, Rochester, New York) and by a radioimmunoassay method using the Linco Human Insulin Specific RIA kit (Linco Research, St. Charles, MO), respectively. A telephone interviewer contacted each participant (or representative) every 6 to 9 months to inquire about hospital admissions, cardiovascular diagnoses and deaths.

### Statistical analyses

Subjects were separated into two genotype groups based on presence or absence of minor allele (G) for all analyses. Categorical variables were summarized as number and percentage and compared between groups using Pearson chi-square test. Continuous variables were examined for normality and presented as mean and standard deviation or median and quartiles, as appropriate. Continuous variables were compared between groups using two-sample t-test or non-parametric Wilcoxon rank-sum test. In order to adjust group comparisons for other covariates such as age, sex, and BMI, regression models were used with adjustment factors as covariates and genotype group was included as an additional covariate. Logistic regression was used to adjust comparisons of dichotomous variables and linear regression was used for continuous variables. Linear regression analysis was carried out using ranks of variables which were not normally distributed.

In order to take into account the effect of lipid lowering agents, lipid values were analyzed in a subgroup of subjects not taking lipid lowering agents. Follow-up endpoints were myocardial infarction, angina pectoris, heart failure, stroke, all-cause mortality and the combined endpoint all cardiovascular diseases including any of the following: myocardial infarction, resuscitated cardiac arrest, angina, stroke, stroke death, coronary heart disease death, other atherosclerotic death, other cardiovascular disease death. They were defined using time to event methodology with censoring at last known status for subjects without events. Cox proportional hazards regression, with adjustment for age and sex, was used to test for association between genotype group and time to event outcomes of interest. Two-sided p-values were used for all analyses and p≤0.005 was defined as statistically significant using a Bonferroni correction based on the number of tests (0.05/10). SAS version 9.4 (Cary, NC) was used for all analyses.

## Results

### Frequencies of rs5068 genotypes and metabolic phenotype

The characteristics of the study population are described in Table 1.

**Table 1:** Characteristics of the study population according to rs5068 genotypes.

CHARACTERISTICS	AA (n = 1520)	AG + GG (n = 110+1)	p-value*	p-value^†^
**Frequency, %**	**93.2**	**6.8**		
**Female**	**813 (53)**	**64 (58)**	**0.39**	**0.40**
**Age, y**	**62 (10)**	**62 (11)**	**0.84**	**0.84**
**Systolic blood pressure, mmHg**	**132 (22)**	**132 (23)**	**0.72**	**0.71**
**Diastolic blood pressure, mmHg**	**75 (10)**	**74 (11)**	**0.53**	**0.62**
Creatinine, mg/dl**	**1.0 (0.9, 1.1)**	**0.9 (0.8, 1.1)**	**0.04**	**0.07**
**Hypertension, n (%)**	**904 (59)**	**59 (53)**	**0.19**	**0.19**
**Body Mass Index, kg/m**^**2**^	**30 (6)**	**30 (6)**	**0.39**	**0.26**
**Obesity, n(%)**	**689 (45)**	**46 (41)**	**0.43**	**0.33**
Waist Circumference, cm**	**100 (91,110)**	**98 (90, 107)**	**0.15**	**0.15**
Total Cholesterol, mg/dl**	**188 (166, 212)**	**185 (167, 208)**	**0.96**	**0.92**
LDL Cholesterol, mg/dl**	**115 (95, 136)**	**113 (94, 137)**	**0.68**	**0.65**
HDL Cholesterol, mg/dl**	**50 (41, 60)**	**54 (44, 65)**	**0.006**	**0.008**
Triglycerides, mg/dl**	**91 (66, 124)**	**80 (62, 113)**	**0.01**	**0.02**
Glucose, mg/dl**^§^	**89 (83, 97)**	**87 (80, 96)**	**0.19**	**0.20**
**Insulin, μU/ml** **^#^	**5.7 (3.6, 8.7)**	**4.8 (3.4, 7.3)**	**0.02**	**0.02**
**Diabetes, n(%)**	**266 (18)**	**9 (8)**	**0.01**	**0.01**
**Metabolic Syndrome, n(%)**	**576 (38)**	**26 (23)**	**0.002**	**0.002**

Values given are n (%) for categorical variables and mean (standard deviation) for continuous variables unless otherwise noted.

*P value obtained from univariate regression analysis.

^†^ P value obtained from regression analysis adjusted for age and sex.

**Variable expressed as median (first quartile, third quartile).

^§^Analysis in a subgroup of subjects with known glucose levels and free of diabetes mellitus (n = 1,351).

^#^Analysis in a subgroup of subjects with known insulin levels and free of diabetes mellitus (n = 1,349). Low density lipoprotein (LDL), high density lipoprotein (HDL).

Genotype frequencies of rs5068 were 93.2% AA (n = 1520), 6.7% AG (n = 110) and 0.1% GG (n = 1). All subsequent analyses were performed by combining AG and GG and comparing with AA. The two groups did not differ in terms of age and sex.

**Fig 1:**
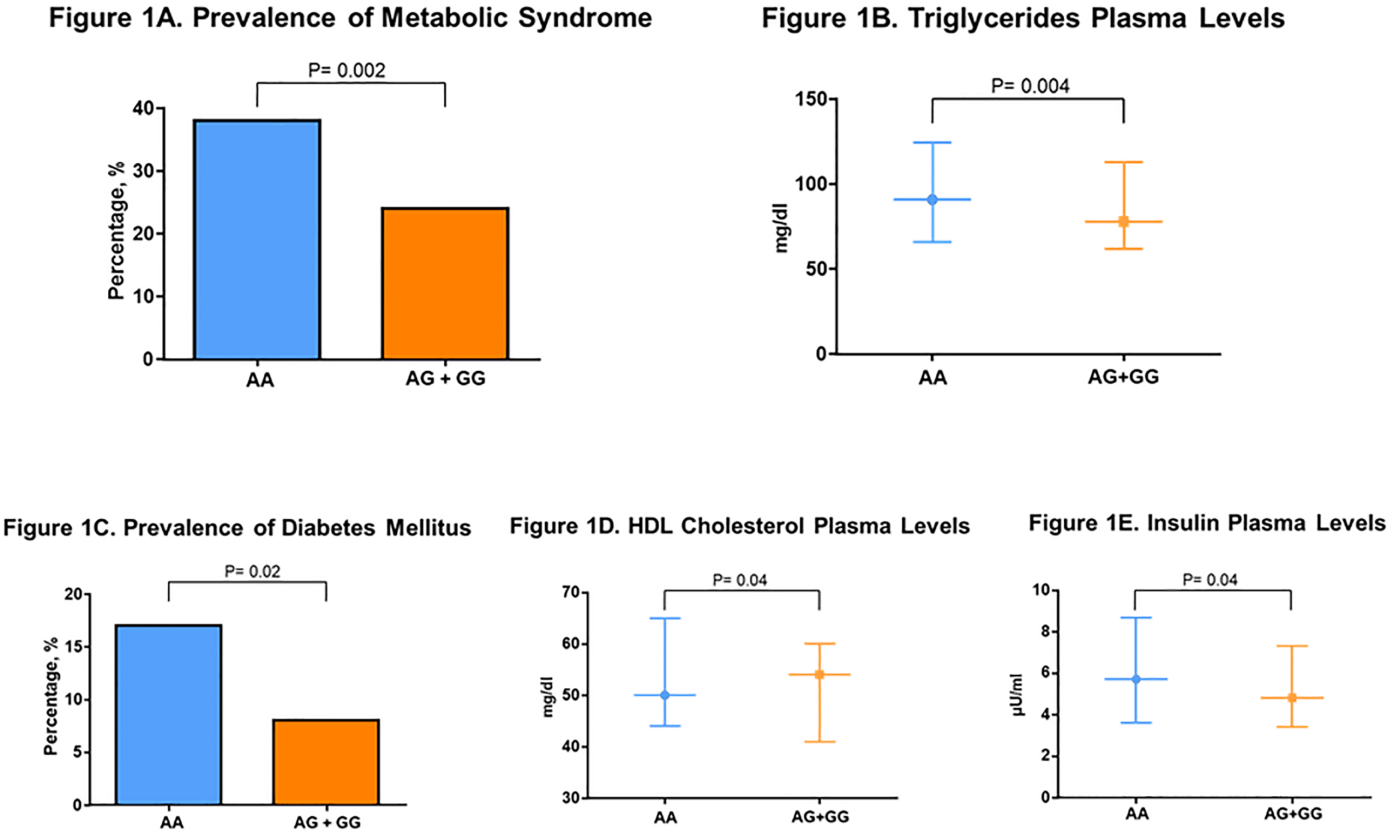
Metabolic phenotype according to rs5068 genotypes. **A. Prevalence of Metabolic Syndrome**. P value obtained from regression analysis adjusted for age and sex. **B. Triglycerides Plasma Levels**. P value obtained from regression analysis adjusted for age, sex and body mass index in a subgroup of subjects not taking lipid-lowering therapy (n = 1370). Values are median, first and third quartile. **C. Prevalence of Diabetes Mellitus**. P value obtained from regression analysis adjusted for age, sex and body mass index. **D. HDL Cholesterol Plasma Levels**. P value obtained from regression analysis adjusted for age, sex and body mass index in a subgroup of subjects not taking lipid-lowering therapy (n = 1370). Values are median, first and third quartile. High-density lipoprotein (HDL). **E. Insulin Plasma Levels**. P value obtained from regression analysis adjusted for age, sex and body mass index in a subgroup of subjects free of diabetes mellitus (AA = 1247, AG+GG = 102). Values are median, first and third quartile.

After applying a Bonferroni correction for multiple testing and using a p-value threshold of 0.005 (0.05/10), the prevalence of metabolic syndrome was significantly lower in the AG+GG genotypes after adjustment for age and sex (23% vs 38%, adjusted p = 0.002) (Fig 1A). In order to account for anti-lipemic treatment we analyzed a subgroup of subjects not taking anti-lipemic medications (AA = 1278, AG+GG = 92) and the carriers of one or two copies of the G allele had significantly lower triglycerides plasma values (76 vs 90 mg/dl, age-sex-BMI adjusted p = 0.004) (Fig 1B). A looser statistical criterion enables identification of additional, non-significant but trending results which are reported as follows. Prevalence of diabetes mellitus tended to be lower in the AG+GG group after adjustment for age, sex and body mass index (8% vs 18%, adjusted p = 0.02) (Fig 1C). When we analyzed the subgroup of subjects that were not on lipid-lowering therapy, HDL-cholesterol levels tended to be higher in the AG+GG group (55 vs 50 mg/dl, age-sex-BMI adjusted p = 0.04) (Fig 1D). Among those subjects not affected by diabetes mellitus, insulin levels tended to be lower in the AG+GG group (4.8 vs 5.7 μU/ml, age-sex-BMI adjusted p = 0.04) (Fig 1E).

Body mass index, waist circumference, prevalence of obesity, plasma values of total and low density lipoprotein cholesterol were similar across genotypes.

### Cardiovascular phenotype

As reported in Table 1, systolic and diastolic blood pressure and prevalence of hypertension did not differ among groups. The use of antihypertensive medications was also similar in the AA and AG+GG genotypes (47% vs 50%, univariate p = 0.13). The multivariate analysis accounting for age, sex, BMI and use of antihypertensive drugs also showed a lack of association between rs5068 genotypes and systolic (adjusted p = 0.39) or diastolic blood pressure (adjusted p = 0.80). In the follow up analysis, the minor allele of rs5068 was not associated with risk for myocardial infarction, angina pectoris, heart failure, stroke, all cardiovascular diseases or all-cause mortality (Table 2).

**Table 2:** Cardiovascular disease and mortality risk according to rs5068 genotypes.

ENDPOINT	AA (n)	AG+GG (n)	Hazard Ratio (95% CI)*	p-value*
**Myocardial Infarction**	**41**	**2**	**0.68 (0.16, 2.79)**	**0.59**
**Angina Pectoris**	**55**	**3**	**0.74 (0.23, 2.37)**	**0.61**
**Heart Failure**	**64**	**2**	**0.42 (0.10, 1.70)**	**0.22**
**Stroke**	**47**	**4**	**1.15 (0.41, 3.18)**	**0.79**
**All Cardiovascular Disease**	**152**	**12**	**1.06 (0.59, 1.92)**	**0.83**
**All-cause Mortality**	**228**	**18**	**1.04 (0.64, 1.69)**	**0.86**

* Cox regression analysis adjusted for age and sex. Confidence interval (CI).

## Discussion

Our study for the first time analyzed the metabolic and cardiovascular phenotype associated with rs5068 genotypes in African Americans, which represent an ethnic group at high risk for cardiovascular and metabolic disease. More specifically, in a community-based cohort of African American individuals residing in 5 different geographic locations in the United States, the AG+GG genotypes of rs5068 are associated with lower prevalence of metabolic syndrome and lower levels of triglycerides. When compared to the AA genotype, the carriers of the minor G allele also tended to have lower plasma values of insulin and higher plasma values of HDL-cholesterol. Prevalence of diabetes mellitus tended to be lower in the AG+GG genotypes. In contrast, the genetic variant rs5068 was not associated with lower systolic or diastolic blood pressure nor was it related with prevalence of hypertension.

Previous studies have shown that in a general community-based cohort from Olmsted County, Minnesota, USA (n = 1,608) the frequencies of rs5068 genotypes were AA: 89.9%, AG: 9.7%, and GG: 0.4% whereas in a random sample of the general population of Ventimiglia di Sicilia, Sicily, Italy (n = 804), genotype frequencies were AA, 93.5%, AG, 6.4% and GG, 0.1% (Table 3).[6, 7]

**Table 3:** Frequencies of rs5068 genotypes.

	AA	AG	GG
**African Americans from MESA**	**93.2%**	**6.7%**	**0.1%**
**(n = 1,631)**	**(n = 1520)**	**(n = 110)**	**(n = 1)**
**Whites from Olmsted County, Minnesota, USA**	**89.9%**	**9.7%**	**0.4%**
**(n = 1,608)**	**(n = 1445)**	**(n = 157)**	**(n = 6)**
**Whites from Ventimiglia di Sicilia, Sicily, Italy**	**93.5%**	**6.4%**	**0.1%**
**(n = 804)**	**(n = 752)**	**(n = 51)**	**(n = 1)**

In these two cohorts of white individuals, the G allele of rs5068 is associated with significantly increased levels of NP and a favorable cardiometabolic profile including lower blood pressure and risk of hypertension, higher plasma values of HDL-cholesterol, lower BMI and waist circumference, decreased risk of obesity and metabolic syndrome. The mechanism which leads to higher ANP levels in the rs5068 G allele carriers was revealed by an elegant study conducted by Arora et al.[21] The G allele prevents the binding of microRNA 425 counteracting the inhibitory action of this microRNA on NPPA expression. In regard to the increased BNP levels associated with rs5068 minor allele, they might be a consequence of the linkage disequilibrium between this genetic variant and other single nucleotide polymorphisms affecting BNP circulating levels.

For the first time our study shows the associations between the rs5068 G allele and a favorable metabolic phenotype in African Americans. These findings are consistent with NP biological properties on lipid and glucose metabolism. Indeed, intravenous infusion of ANP in healthy individuals induces lipid mobilization and oxidation in adipose tissue and increases post-prandial energy expenditure.[22, 23] Moreover, activation of the NP system enhances mitochondrial oxidative metabolism in human skeletal muscle.[24] In mice, overexpression of the BNP gene conferred protection from high-fat diet induced obesity and insulin resistance.[2] The evidence of an interaction between NP and insulin is also provided by the study of Pivovarova et al. in which the expression of the NP clearance receptor is upregulated by insulin in the subcutaneous adipose tissue of obese individuals.[25] Insulin also induces the expression of the NP clearance receptor gene in visceral human adipocytes through a glucose-dependent modulation.[4] Indeed, low-glucose medium, which simulates starving conditions, decrease the insulin mediated upregulation of the NP clearance receptor. In addition, Coue’ et al demonstrated that in human skeletal muscle GC-A expression positively correlates with insulin sensitivity whereas the expression of the NP clearance receptor is inversely related to this metabolic parameter.[26] Moreover, chronic infusion of BNP improved glucose tolerance and peripheral insulin sensitivity in a rodent model of obesity and diabetes. Several epidemiologic studies further support the link between NP and metabolic profile. Circulating levels of NP are inversely related to BMI and they are lower in obese subjects and diabetic patients.[27–29] Atrial natriuretic peptide and BNP levels are decreased in individuals with metabolic syndrome and low plasma levels of NP are predictive of future development of diabetes.[28, 30, 31] Interestingly, the minor alleles of the genetic variants rs5068 and rs198389 that are associated with increased levels of ANP and BNP are also associated with lower risk of diabetes mellitus in whites.[9, 32]

Importantly, the frequencies of the rs5068 AG+GG genotypes observed in African Americans are similar to the ones previously reported in a Sicilian cohort but they are lower when compared to whites from Olmsted County, Minnesota. Despite slightly different frequencies in different ethnic groups, the rs5068 G allele is associated with a favorable cardiovascular and metabolic profile in whites and a favorable metabolic profile in African Americans. Indeed, in our study, the rs5068 genotypes did not correlate with blood pressure and hypertension. One possible explanation could be related to the relative deficiency of the nitric oxide system and exaggerated activation of the renin-angiotensin-aldosterone system that characterize African Americans.[33–35] Among this ethnicity, slightly higher values of NP like the ones observed in the carriers of the rs5068 minor allele may impact metabolism and confer a favorable metabolic risk profile but they might not be sufficient to counteract deficient nitric oxide or inappropriately augmented aldosterone actions for which a stronger activation of the NP system might be required. Conversely, another possible explanation for the lack of association could be related to the selection criteria of MESA which excluded any subject with clinically apparent cardiovascular disease at baseline. Consequently, the characteristics of the study cohort might have affected our analysis of the relationship between rs5068 genotypes and blood pressure. In addition, the number of subjects with AG+GG genotype is relatively small (n = 111), and thus power was limited to detect an association between rs5068 genotypes and blood pressure. Nonetheless, the size of this study cohort was comparable to the one of our previous reports in whites. [6, 7] Moreover, rs5068 genotypes were not associated with any of the cardiovascular clinical outcomes evaluated in our follow-up analysis but the power for our analysis was limited by the small numbers of events in the AG+GG group. Additional studies in larger African American cohorts are needed to further evaluate the associations between rs5068 genotypes and the cardiovascular phenotype.

In consideration that the characteristics of the cardiometabolic phenotype can be interrelated in regard to genotype, in our statistical analysis, we assessed the relationship between rs5068 genotypes and each characteristic separately.[36] Nevertheless, we recognized the possibility of false positive results and presented a p-value threshold corrected for multiple testing. After Bonferroni correction the G allele of rs5068 was associated with prevalence of metabolic syndrome and triglycerides levels in subjects not taking anti-lipemic medications. Considering that the diagnosis of metabolic syndrome includes already by itself the presence of altered glucose and/or lipid metabolism, the significant association between rs5068 G allele and this cardiometabolic disease in addition to the significant relationship with tryglicerides levels further supports the hypothesis of a key role played by NP in the cardiometabolic clinical phenotype and risk. Future studies in larger cohorts are warranted to further investigate the metabolic phenotype associated with rs5068 genotypes in African Americans.

Our study has limitations. In MESA, circulating levels of the biologically active ANP and BNP were not measured whereas the circulating levels of the non-biologically active N-terminal-pro-BNP have been previously assessed. Importantly, N-terminal-pro-BNP levels inversely correlated with BMI, triglycerides and insulin resistance whereas they were positively associated with HDL-cholesterol levels.[19] As shown by previous studies discussed above, plasma ANP and BNP concentrations are higher among rs5068 minor allele carriers in general communities of European ancestry.[5, 6] The favorable metabolic phenotype observed in our African American cohort support the hypothesis that rs5068 minor allele might be associated with higher circulating levels of NP in African American individuals as well. Consistent with our results, Neeland et al reported that higher BNP levels are associated with a healthier body fat profile in the Dallas Heart Study cohort, which includes mostly blacks.[37]

The current study adds significantly to our understanding of the clinical genomics of the NP system with special insight into racial differences and therapeutic implications. Now across three different general populations and two ethnic groups (whites in North America and Sicily and now African Americans in the United States), rs5068 G allele is associated with a specific metabolic phenotype. This metabolic phenotype is characterized by a favorable cardiometabolic risk profile and more specifically, reduced incidence of metabolic syndrome and obesity with a favorable lipid and insulin profile. As we consider clinical and therapeutic implications, NP-like based therapeutics may represent an opportunity to target cardiometabolic disease across ethnic groups.

## Supporting information

S1 File 2017.11.14_Supporting_Information.docx. The file defines the availability of the MESA dataset.
(DOCX)

## Abbreviations

ANP: Atrial natriuretic peptide
BMI: body mass index
BNP: B-type natriuretic peptide
cGMP: cyclic guanosine monophosphate
GC-A: particulate guanylyl cyclase-A receptor
HDL: high-density lipoprotein
MESA: Multi-Ethnic Study of Atherosclerosis
NP: natriuretic peptides
NPPA: atrial natriuretic peptide gene
