# Supplementary material for: A favorable cardiometabolic profile is associated with the G allele of the genetic variant rs5068 in African Americans: The Multi-Ethnic Study of Atherosclerosis (MESA)
Source: PLoS One. 2017 Dec 18;12(12):e0189858. doi: 10.1371/journal.pone.0189858 (PMC5734753; doi:10.1371/journal.pone.0189858)
Supplement: S1 File — The file defines the availability of the MESA dataset. (DOCX) [file pone.0189858.s001.docx]

**Supporting information**

The MESA (<https://www.mesa-nhlbi.org/>) dataset was obtained from the MESA coordinating center at Mayo Clinic and was analyzed at Mayo Clinic. Availability of the MESA dataset is controlled by the MESA coordinating center (<https://biolincc.nhlbi.nih.gov/studies/mesa/>).
